# Measures for the Prevention of Mother-to-Child Human T-Cell Leukemia Virus Type 1 Transmission in Japan: The Burdens of HTLV-1-Infected Mothers

**DOI:** 10.3390/v15102002

**Published:** 2023-09-26

**Authors:** Kaoru Uchimaru, Kazuo Itabashi

**Affiliations:** 1 Department of Tumor Cell Biology, Graduate School of Frontier Sciences, The University of Tokyo, Tokyo 1088639, Japan; 2 Aiseikai-Memorial Ibaraki Welfare and Medical Center, Ibaraki 3100836, Japan; kitaba@med.showa-u.ac.jp

**Keywords:** human T-cell leukemia virus, mother-to-child transmission, exclusive formula feeding, short-term breastfeeding

## Abstract

The main mode of mother-to-child transmission of the human T-cell leukemia virus (HTLV)-1 is through breastfeeding. Although the most reliable nutritional regimen to prevent HTLV-1 transmission is exclusive formula feeding, a recent meta-analysis revealed that short-term breastfeeding within 90 days does not increase the risk of infection. The protocol of the Japanese Health, Labor, and Welfare Science Research Group primarily recommended exclusive formula feeding for mothers who are positive for HTLV-1. However, there has been no quantitative research on the difficulties experienced by HTLV-1-positive mothers in carrying out these nutritional regimens, including the psychological burden. Therefore, this review was performed to clarify the burdens and difficulties encountered by mothers who are positive for HTLV-1; to this end, we analyzed the data registrants on the HTLV-1 career registration website “Carri-net” website. The data strongly suggest that it is not sufficient to simply recommend exclusive formula feeding or short-term breastfeeding as a means of preventing mother-to-child transmission; it is important for health care providers to understand that these nutritional regimens represent a major burden for pregnant women who are positive for HTLV-1 and to provide close support to ensure these women’s health.

## 1. Introduction

Human T-cell leukemia virus (HTLV)-1 is a human retrovirus responsible for a number of associated diseases, including adult T-cell leukemia lymphoma (ATL), HTLV-1-associated myelopathy (HAM), and HTLV-1-associated uveitis (HAU) [1,2,3]. ATL is an intractable hematological malignancy that occurs in about 5% of infected individuals, and the acute form of which has a median survival time of only 252 days in Japan [4,5]. HAM is a spinal inflammatory disease with an incidence of about 0.3% of infected individuals, which causes spastic paraparesis of the lower limbs and urinary dysfunction [2]. Although not life-threatening, HAM has a significant adverse effect on the activities of daily living (ADL) of patients, and no satisfactory treatment is currently available [6]. As there are no effective treatments for these diseases, it is important to prevent HTLV-1 infection. Based on blood donor data, the number of HTLV-1 carriers in Japan is estimated to be about 6–700,000 [7], but this may be an underestimate as seroprevalence rates estimated from blood donation data tend to be much lower than from antenatal screening data [8]. The prevalence of HTLV-1 worldwide is estimated to be 5–10 million, but again, this is likely an underestimate. HTLV-1 is endemic in several areas, including sub-Saharan Africa, New Guinea, the Caribbean islands, central Australia, and southwestern Japan [9].

HTLV-1 infection occurs through three main routes, i.e., breastfeeding from an infected mother, sexual intercourse, and blood transfusion [10]. HTLV-1 antibody screening of donated blood began in 1986 in Japan, and there is no infection by blood transfusion in the country. Avoiding breastfeeding by HTLV-1-infected mothers is an effective means of preventing mother-to-child transmission through breast milk [11,12,13], but several observational studies have reported that breastfeeding within 90 days and feeding with frozen/thawed breast milk did not increase the infection rate among infants [14]. In Japan, anti-HTLV-1 antibody screening has been performed at antenatal checkups for all pregnant women at public expense since 2010 [15]. Pregnant women who are positive for anti-HTLV-1 antibodies were advised to select one of the three feeding protocols for their infants: exclusive formula feeding, short-term breastfeeding within 90 days, and use of frozen/thawed breast milk. The most reliable regimen to prevent mother-to-child transmission is exclusive formula feeding, because babies never take breast milk. However, the evidence for the effectiveness of preventing mother-to-child HTLV-1 transmission is insufficient for short-term breastfeeding because of the small sample sizes in previous studies. Therefore, the Japanese nutritional regimen for HTLV-1-infected mothers was changed in 2017 to recommend exclusive formula feeding as the first choice among these three regimens [16]. Pregnant women in Japan are now recommended to prefer exclusive formula feeding over short-term breastfeeding. Nevertheless, if they desire to breastfeed, they are advised to choose short-term breastfeeding. Breastfeeding has many advantages over formula feeding for the health and development of the child and for the health of the mother [17]. Therefore, it is the best nutritional regimen for infants, and there is a general trend toward breastfeeding for infants in Japan. Accordingly, it is anticipated that pregnant women diagnosed as HTLV-1 carriers will face significant conflicts in choosing the method for feeding their babies. Although it is very important to assess the burden of HTLV-1-infected mothers in choosing a nutritional regimen for their babies to allow the provision of optimal support, little is known about this issue.

This survey on the thoughts and feelings of HTLV-1-infected mothers in Japan was performed based on data from the HTLV-1 carrier registration website Carri-net (https://htlv1carrier.org, accessed on 11 September 2023) which we built and have operated since 2015. These data are expected to be important for building a support system for mothers diagnosed as carriers of HTLV-1.

## 2. Materials and Methods

### 2.1. Website and Data Collection

Carri-net is a website for voluntary registration of HTLV-1-infected individuals constructed in 2015 and supported by a Health and Labor Science Research Grant (Grant Number H26-ganseisaku-ippan-006). Registrants answer questions about how their infection was discovered, subsequent actions, and so forth on registration, to allow the collection of information on the actual status of HTLV-1-infected patients in Japan by tabulating the various background factors. Starting in 2017, a secondary survey was conducted on the website for registrants who had experienced pregnancy and/or childbirth (Table 1).

A third survey regarding whether mothers who were HTLV-1 carriers would prefer short-term breastfeeding within 90 days if it would not increase the risk of infection in the child was begun in December 2022. The questionnaire for registrants who had experienced pregnancy and/or childbirth included information outlining that research shows no increase in the infection rate of infants by short-term breastfeeding for 90 days or less compared to exclusive formula feeding, and it also asked whether they would prefer short-term breastfeeding within 90 days if it has no impact on the infection rate in infants.

Collected data were fixed in February 2022 for the second survey and February 2023 for the third survey.

### 2.2. Institutional Review

This study was approved by the institutional review board of the University of Tokyo (approval number 18–36) and performed in accordance with the tenets of the Declaration of Helsinki.

## 3. Results

### 3.1. Registrants

In total, 768 individuals with HTLV-1 infection registered on Carri-net, and 256 individuals who had experienced pregnancy and/or childbirth responded to the second survey. The percentage of those diagnosed during antenatal checkups was 67.7%, while 20.1% were diagnosed as infected with HTLV-1 at a checkup on blood donation before pregnancy. The date of last birth was before March 2011 in 46.1% of registrants, between April 2011 and March 2017 in 27.3%, and after April 2017 in 25.8%. The third survey, which began in 2022, had 78 respondents. The percentages of those diagnosed during antenatal checkups and at blood donation were 61.0% and 28.6%, respectively. The proportion of those diagnosed during antenatal checkups was almost the same in each survey.

### 3.2. Choice of Postnatal Nutritional Regimen

**Figure 1:**
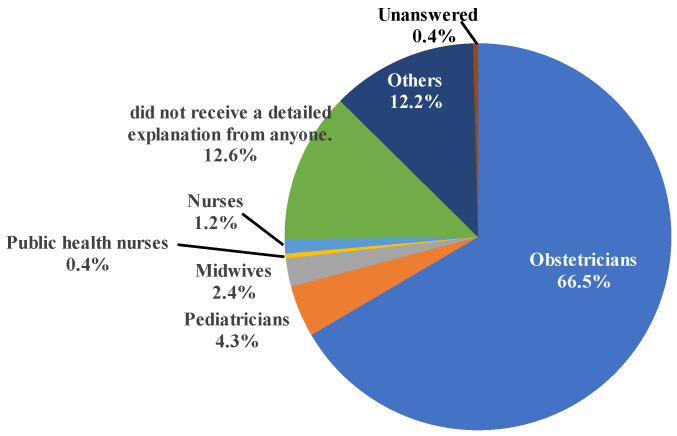
Health care providers who explained about mother-to-child transmission to HTLV-1-carrier mothers. The majority of health care providers who provided explanations regarding mother-to-child HTLV-1 transmission and prevention methods were obstetricians and gynecologists.

The majority of health care providers who provided explanations regarding mother-to-child HTLV-1 transmission and prevention methods were obstetricians and gynecologists (66.5%) followed by pediatricians (4.3%) (Figure 1). Only 2.4%, 1.2%, and 0.4% of respondents answered that such explanations were provided by midwives, nurses, and public health nurses, respectively. On the other hand, 10.2% of the respondents indicated that they did not receive any such explanations.

**Figure 2:**
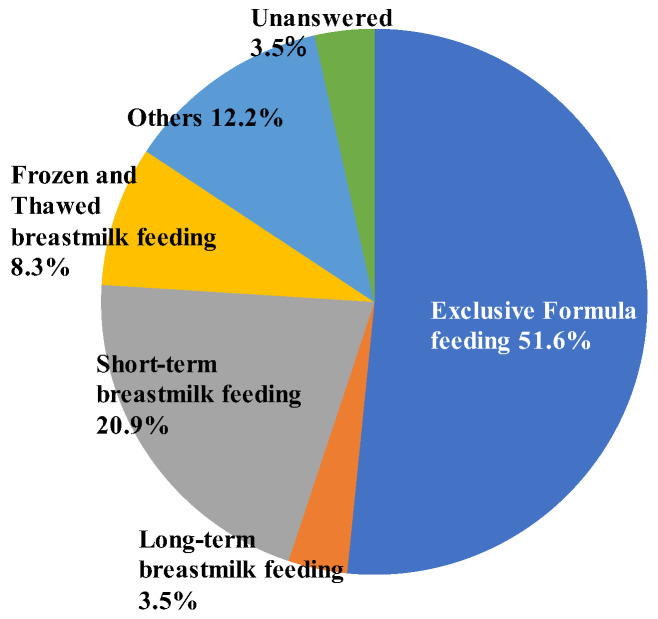
Nutrition protocol chosen by HTLV-1-infected mothers. Aggregated counts of all feeding period are shown in this figure.

**Figure 3:**
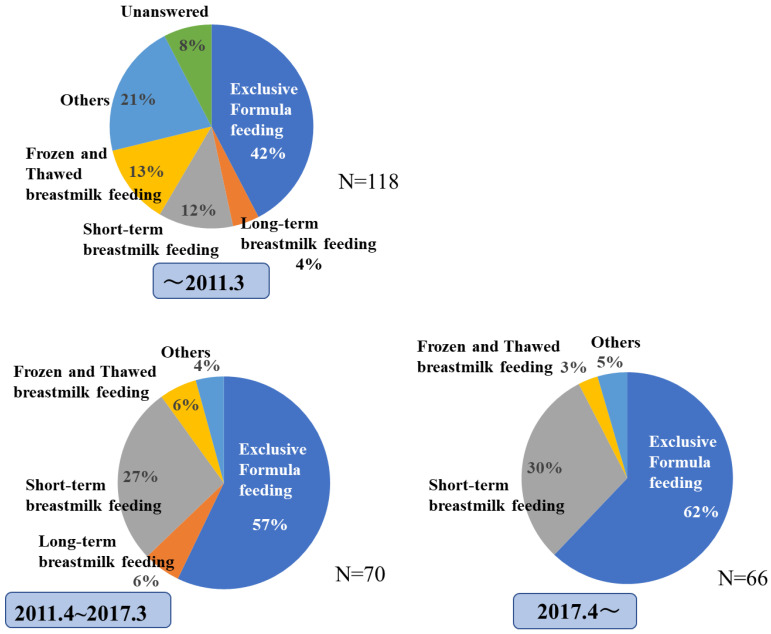
Nutrition protocol chosen by HTLV-1-infected mothers. The data were separated into 2011 and before, 2011–2017, and 2017 according to the time of lactation. Nutritional methods selected varied depending on when they were implemented. The percentage of exclusive formula feeding increased from before March 2011 to after April 2017. On the other hand, the proportion of short-term breastfeeding was also increasing.

The proportions of HTLV-1-infected mothers who chose each nutritional regimen are shown in Figure 2. Exclusive formula feeding was chosen by 51.6% of HTLV-1-infected mothers, while short-term breastfeeding (within 90 days) was chosen by 20.9%, and frozen/thawed breast milk was chosen by 8.3%. A few chose long-term breastfeeding (longer than 90 days). The proportions of each nutritional regimen chosen by HTLV-1-infected mothers changed between the different periods examined. That is, the percentage of exclusive formula feeding was 42% before March 2011, which changed to 57% between April 2011 and March 2017 and 62% after April 2017. On the other hand, the proportion of short-term breastfeeding increased from 12% before 2011 to 27% between 2011 and 2017, increasing further to 30% after 2017 (Figure 3). There were no HTLV-1 carriers who chose long-term breastfeeding among the respondents after 2017.

### 3.3. Thoughts of HTLV-1-Carrier Mothers on Nutrition for Their Infants

The mothers who were HTLV-1 carriers were asked whether the regimen they had chosen was difficult. Overall, 37% of the respondents indicated that it was difficult to follow the chosen nutritional regimen regardless of the actual regimen used. Those who indicated that it was difficult were then asked to specify the reason why they felt this way. As shown in Table 2, the most common response was that they felt guilty for not breastfeeding (74.1%) followed by that not breastfeeding made them feel less valuable (45.9%). Other factors cited by around 20% of the HTLV-1-carrier mothers were the difficulty of interrupting breastfeeding, the complexity of freezing and thawing breast milk, and the lack of support from health care providers. Some mothers cited insufficient family cooperation.

Next, the respondents were asked whether they had received sufficient support from their health care providers regarding the prevention of mother-to-child HTLV-1 transmission during the course of pregnancy, delivery, and child rearing. About two-thirds of the respondents (67%) responded that they had received insufficient support. As shown in Table 3, the two most frequently mentioned issues were insufficient information about where they could receive support about their concerns (51.9%) and inadequate understanding and an insufficient response to their thoughts and feelings by medical professionals (47.4%). Other issues mentioned were that health care providers were not familiar with mother-to-child HTLV-1 transmission, there was little or no linkage between obstetrics and gynecology and pediatrics, insufficient explanation of mother-to-child HTLV-1 transmission, and inadequate support for specific nutritional regimens.

**Figure 4:**
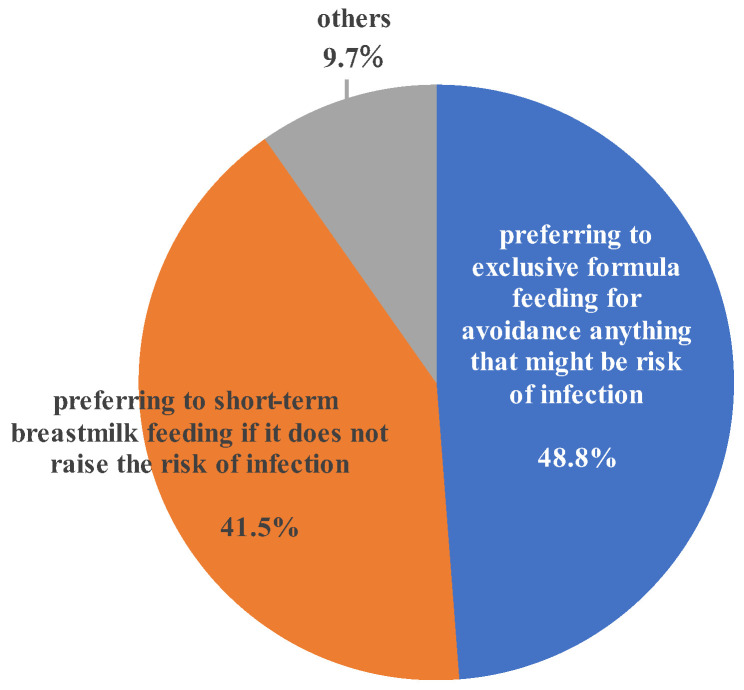
Preference of nutrition protocol assuming that short-term breastmilk feeding does not raise the possibility of HTLV-1 infection to the babies. About half of the HTLV-1-carrier mothers choose exclusive formula feeding even if short-term breastmilk feeding has no impact on the infection rate in infants for fear of infection risk.

In the third survey begun 5 years after commencement of the second survey, 42.9% of respondents indicated that they would prefer short-term breastfeeding within 90 days if it would not increase the risk of infection of their infants compared to exclusive formula feeding, while 48.1% responded that they would prefer exclusive formula feeding due to a fear of even low infection risk (Figure 4).

## 4. Discussion

The main modes of HTLV-1 transmission are vertical mother-to-child transmission and horizontal transmission through sexual intercourse [10]. Since the late 1980s, it has been reported that the major route of mother-to-child transmission has been through breastfeeding [11,12,13,14,18]. The ATL prevention program (APP) in Nagasaki Prefecture showed that the rate of HTLV-1 infection in the babies of pregnant women who were HTLV-1 carriers was around 20%, but this was reduced to about 3% by exclusive formula feeding [11]. Based on these results, exclusive formula feeding was recommended for mothers infected with HTLV-1, particularly in Nagasaki prefecture in Japan. Several studies supported by the Ministry of Health, Labor, and Welfare of Japan and several other investigations have reported that there are no significant differences in the infection rate of babies of HTLV-1-infected mothers between those with short-term breastfeeding within 90 days and exclusive formula feeding [19]. Therefore, short-term breastfeeding has been one of the recommended nutritional regimens for mothers infected with HTLV-1 since 2011 in Japan [20]. Short-term breastfeeding can provide the partial benefits of breast milk, including nutritional and immunological benefits, prevention of many diseases, promotion of mother–child bonding, and maternal recovery after delivery, and mothers can have the satisfaction of feeding their infants with their own breast milk. In this study, the proportion of HTLV-1-carrier mothers who chose short-term breastfeeding increased from 12% before 2011 to 30% after 2017, along with an increase in the proportion of those choosing exclusive formula feeding from 42% before 2011 to 62% after 2017. After 2011, long-term breastfeeding was chosen only rarely (Figure 2 and Figure 3). However, there was insufficient evidence for a lack of risk for mother-to-child transmission of HTLV-1 by short-term breastfeeding at that time. This is due to the small sample sizes of previous studies and the fact that, theoretically, the most reliable way to prevent mother-to-child transmission is to avoid breastfeeding. Hence, the recommended nutritional regimen for HTLV-1-carrier mothers was changed to exclusive formula feeding, and short-term breastfeeding was limited to mothers who nevertheless desired to breastfeed after 2017 [15]. However, it is an important fact that the proportion of carrier mothers who chose short-term breastfeeding did not decrease after 2017, despite the change in the recommendations (Figure 3). Due to the various benefits of breast milk, breastfeeding is generally recommended, and there is a general trend that babies should be breastfed in Japan. This tends to cause feelings of guilt in mothers for not breastfeeding and to choose breastfeeding if possible, even if only for a short time. As shown in Table 2, 37% of the carrier mothers indicated that they felt the chosen nutritional regimen was difficult, and the most common reasons were that they felt guilt (74.1%) or felt less valuable (45.9%).

Miyazawa et al. conducted a prospective cohort study and meta-analysis to determine the efficacy of short-term breastfeeding to prevent mother-to-child transmission of HTLV-1 beginning in 2012, before the change in nutritional regimen recommendations [19]. They enrolled 735 mothers with confirmed HTLV-1 infection over a period of 3 years and followed up 313 babies born to these women. The proportions of mothers who chose exclusive formula feeding, short-term breastfeeding, frozen/thawed breastfeeding, and long-term breastfeeding were 35.1%, 55.0%, 6.1%, and 3.8%, respectively. The percentage of infected babies who were fed by short-term breastfeeding was 2.3%, which was not significantly different from the rate of those with exclusive formula feeding (6.4%). In addition, they performed a systematic review and meta-analysis including their own results and reported a relative HTLV-1 infection risk of 1.14 for babies given short-term breastfeeding within 3 months compared to those given exclusive formula feeding (95% CI: 0.20–6.50; *p* = 0.88). Those results suggest that short-term breastfeeding did not increase the risk for HTLV-1 infection compared to exclusive formula feeding.

Another important result of their study was that a considerable proportion of carrier mothers who chose short-term breastfeeding could not stop breastfeeding within 3 months, i.e., 33.5% of HTLV-1-positive mothers continued breastfeeding at 3 months after delivery, and 8% continued for 6 months. The longitudinal change in percentage of babies fed breast milk whose mothers chose short-term breastfeeding was estimated by second-order polynomial analysis, and the results indicated that the rate of breastfeeding in this group was 18.2% at 4 months after delivery. Therefore, it was difficult for mothers who chose short-term breastfeeding to terminate this feeding regimen. There are many possible reasons for this difficulty, including when the baby does not take milk after switching to a bottle and mothers continue to breastfeed out of guilt, and mothers lack access to adequate breast care. In some cases, the desire to allow the baby to feed from their own breast had become so strong that they felt they could not stop. Therefore, a variety of support is needed for mothers to achieve successful short-term breastfeeding.

Based on these results, the nutritional recommendations were changed again in 2022. This manual for prevention of mother-to-child transmission of HTLV-1 by the Health, Labor, and Welfare Science Research Group in Japan specifies that the selection of nutritional regimen by HTLV-1-infected mothers should be accompanied by sufficient explanation of the advantages and disadvantages of each nutritional regimen from the perspective of pregnancy, childbirth, and childcare, in addition to the prevention of mother-to-child transmission and shared decision-making support [21] so that mothers can make their own choices. Based on these principles, exclusive formula feeding is recommended as the regimen with the most established evidence, but short-term breastfeeding can be chosen if desired, with a transition to exclusive formula feeding within 90 days. In the process of selecting a nutritional regimen and carrying it out, mothers and health care providers are required to engage in evidence-based support and narrative-based, interactive communication. Health care providers who provide support should understand the difficulties associated with these decisions and respond appropriately, as there are common and characteristic difficulties regardless of which nutritional method is chosen.

As shown by Miyazawa et al. [19], it is difficult for HTLV-1-carrier mothers who chose short-term breastfeeding to stop breastfeeding and change to exclusive formula feeding before 90 days after delivery without support from health care providers. Mothers may face technical difficulties in breast care and transitioning from breastfeeding to exclusive formula feeding, as well as psychological distress. Technical and psychological support for HTLV-1-carrier mothers by midwives and other health care professionals is essential for successful short-term breastfeeding [22]. Short-term breastfeeding is listed as one of the nutritional options for HTLV-1-infected mothers along with exclusive formula feeding in the revised recommendations published in 2022, but it is clearly stated that it is essential to provide support, including appropriate breast care, at midwife and lactation support outpatient clinics, as termination of breastfeeding and transition to full artificial nutrition by 90 days of age is expected to be accompanied by various difficulties. Different types of support should be offered to pregnant women confirmed to be carriers of HTLV-1, including those who choose exclusive formula feeding.

HTLV-1-infected mothers have various burdens regardless of the selected nutritional regimen. In this study, 37% of the responders indicated that it was difficult to follow the selected nutritional regimen. As shown in Table 2, the most frequent reason was feeling guilty for not breastfeeding (74.1%), followed by feeling less valuable (45.9%). As all responses of carrier mothers who chose each nutritional regimen were combined and over half of the responders (51.6%) chose exclusive formula feeding, it is assumed that these results strongly reflect the thoughts and feelings of the mothers who chose exclusive formula feeding. Our results strongly suggest that it is not sufficient to allow HTLV-1 carrier pregnant women to choose between exclusive formula feeding and short-term breastfeeding as mother-to-child HTLV-1 transmission prevention measures; health care providers must keep in mind that this represents a major conflict for these women. However, 67% of the responders indicated that the support of health care providers regarding the prevention of mother-to-child HTLV-1 transmission during pregnancy, delivery, and child rearing was insufficient. As shown in Table 3, 47.4% of the HTLV-1-infected mothers who indicated that support from health care providers was insufficient reported that medical professionals did not necessarily understand and respond to their thoughts and feelings. Choosing exclusive formula feeding to prevent HTLV-1 infection of the child is a serious decision for the mother, and health care providers are expected to support the mother’s decision to choose exclusive formula feeding and be attentive to their thoughts and feelings.

Next, we asked HTLV-1-positive registrants who had experienced pregnancy and/or childbirth whether they would prefer short-term breastfeeding within 90 days if it would not increase the infection risk in their infants compared to exclusive formula feeding after providing an explanation that research data support this view before the recommendation of nutrition protocol was revised. As shown in Figure 4, 42.9% of HTLV-1-infected mothers indicated that they would choose short-term breastfeeding if it would not increase the risk for mother-to-child transmission, while 48.1% indicated that they would still prefer exclusive formula feeding as it is the most reliable means of preventing mother-to-child transmission through breastfeeding and would not do anything that may pose a risk of infection in their infants even if the risk was low. It is assumed that they would choose exclusive breastfeeding for the sake of their babies, despite wanting to breastfeed. It is also necessary to understand the strong desire of HTLV-1-carrier mothers to prevent transmission of the virus to their babies and to be mindful of the emotional burden that they bear because of their choice.

On the other hand, 51.9% of the mothers who responded that the support of health care providers was insufficient indicated that they had insufficient information about who to consult about their concerns, including those about HTLV-1-associated diseases (Table 3). A diagnosis of positivity for anti-HTLV-1 antibodies means that they are HTLV-1 carriers and at risk for developing HTLV-1-associated diseases in the future, such as ATL, so they wished to know about these diseases along with methods of preventing transmission of the virus to their babies. As ATL and HAM are hematological and neurological diseases, respectively, while HAU is an ophthalmological disease, HTLV-1-carrier mothers cannot consult with obstetricians regarding these diseases. Moreover, they are relatively rare, and, for example, not all hematologists can provide thorough explanations of ATL. Therefore, it is necessary to establish a systematic system of consultation with specialists. This is also true between obstetricians and pediatricians (Table 3). HTLV-1-carrier mothers have various concerns about their children during the child-rearing process, and so it is desirable for obstetricians to collaborate with pediatricians regarding follow-up of the children. Carrier mothers were not satisfied with the lack of collaboration among obstetricians, pediatricians, hematologists, neurologists, and local government officials.

Recently, the number of HTLV-1 infectants through horizontal transmission in Japan was evaluated by Satake et al. [23]. They assessed blood donors who became positive for anti-HTLV-1 antibodies despite previous negative results over a 4.5-year period and estimated that more than 4000 individuals were newly infected by HTLV-1 each year in Japan. On the other hand, the incidence of mother-to-child HTLV-1 transmission was estimated based on the number of pregnant women positive for anti-HTLV-1 antibodies in antenatal screening. The Japan Society of Obstetricians and Gynecologists estimates the number of carrier mothers to be about 1200 per year in Japan [24]. The majority of pregnant women with HTLV-1 infection choose exclusive formula feeding or short-term breastfeeding in Japan [19], and so the rate of infection among their babies would be around 3%. Consequently, the annual number of newly HTLV-1-infected babies is around 30 or 40 in Japan. Therefore, the main route of HTLV-1 transmission in Japan is now horizontal transmission, mainly through sexual intercourse. Nevertheless, it is important to prevent mother-to-child transmission because most cases of ATL are attributable to this route of infection [4,25], and ATL is an intractable and fatal hematological malignancy [5]. On the other hand, methods of prevention, such as exclusive formula feeding or short-term breastfeeding, have various drawbacks, including the inability to obtain the benefits of breast milk, the psychological burden on carrier mothers, and economic issues such as the cost of purchasing formula, particularly in developing countries. About 80% of the babies of HTLV-1-infected mothers do not become infected with the virus even without precautions to prevent infection through breast milk, and as a result, precautionary measures were not necessary for these mothers. It is necessary to identify reliable predictors of the risk of mother-to-child transmission, as the benefits and drawbacks of breastmilk infection prevention measures need to be thoroughly considered.

## 5. Conclusions

The most reliable nutritional regimen to prevent mother-to-child HTLV-1 transmission is exclusive formula feeding, but short-term breastfeeding within 90 days does not increase the risk of infection among babies of HTLV-1-infected mothers. Although short-term breastfeeding can provide partial benefits of breast milk, it should be noted that it is not always easy to terminate breastfeeding within 90 days, and a suitable support system is essential. On the other hand, exclusive formula feeding represents a critical choice for HTLV-1-carrier mothers and places a psychological burden on these women. It is necessary to provide not only technical but also psychological support for these mothers. It should be noted that HTLV-1-carrier mothers carry a variety of burdens.

## Figures and Tables

**Table 1:** Questions for registrants who have experienced pregnancy or childbirth.

(1) What is the date of birth of your child? a. Before 31 March 2011. b. Between 1 April 2011 and 31 March 2017. c. On or after 1 April 2017.
(2) At what time were you diagnosed as an HTLV-1 carrier? a. In this pregnancy. b. In a previous pregnancy. c. During a blood donation. d. Other
(3) Which health care providers mainly provided explanations about mother-to-child HTLV-1 transmission and prevention methods? a. OB/GYN physician. b. Pediatrician. c. Midwife. d. Public health nurse. e. Nurse. f. I did not receive a detailed explanation from anyone. g. Other.
(4) Which method of breastfeeding did you choose before delivery to prevent mother-to-child transmission of infection? a. No breast milk at all (formula). b. Breastfeeding for as long as possible without limitation (long-term breastfeeding). c. Breastfeeding within the first 3 months of life and then formula (short-term breastfeeding). d. Breast milk was frozen and then thawed (frozen/thawed breast milk). e. Other.
(5) Whose opinion did you consult most in choosing breastfeeding before delivery? a. Health care provider. b. Husband or partner. c. Own mother or parents. d. Others.
(6) Was your choice of breastfeeding method easy? a. Easy. b. Not easy.
(7) If you answered “b. Not easy” in question (6), please answer the following. What were the difficulties? (multiple responses are acceptable.) a. It was difficult to stop breastfeeding. b. Freezing and thawing breast milk was complicated. c. People around me pointed out that I was using artificial nutrition. d. I felt guilty for not being able to breastfeed. e. Insufficient support from medical personnel. f. Lack of cooperation from family members. g. Other.
(8) Do you think the support of medical personnel regarding mother-to-child HTLV-1 transmission and its prevention during pregnancy, delivery, and child rearing is sufficient? a. Adequate. b. Inadequate.
(9) If you answered “b. Inadequate” in question (8), what do you think of the following points? What are the main reasons for this? (multiple answers are acceptable.) a. Insufficient explanation about prevention of mother-to-child transmission. b. Health care providers are not familiar with mother-to-child HTLV-1 transmission. c. Specific nutritional support is needed. d. I would like guidance that is sensitive to the mother’s feelings. e. There is little cooperation between obstetrics and gynecology and pediatrics. f. I did not know where to consult. g. Other.

**Table 2:** The reason why HTLV-1-carrier mothers feel that the nutrition protocol chosen by them was difficult. Eighty-five responders who indicated that the nutrition protocol they chose was difficult were asked the reasons. Multiple responses were allowed.

Reason	Responders	%
Stopping breastmilk feeding is difficult.	20	23.5
Freezing and thawing of breast milk was complicated.	19	22.4
People around me pointed out that I was on artificial nutrition, and I felt less valuabale.	39	45.9
Guilt over not breastfeeding.	63	74.1
Insufficient support from medical personnel.	14	16.5
Family members were not cooperative.	3	3.5
Others.	23	27.1
Total responders.	85	100%

**Table 3:** The reason why HTLV-1-carrier mothers indicated the support of health care providers regarding prevention of HTLV-1 mother-to-child transmission. One hundred and fifty-four responders who indicated it was insufficient were asked the reasons. Multiple responses were allowed.

Reason	Responders	%
insufficient explanation of prevention of mother-to-child transmission	43	27.9
health care providers are not familiar with HTLV-1 mother-to-child transmission	57	37.0
lack of assistance with specific nutritional methods.	39	25.3
I would like to be guided by the mother’s feelings	73	47.4
little or no linkage from obstetrics to pediatrics	53	34.4
little information about where to go for advice	80	51.9
others	26	16.9
total	154	100

## Data Availability

The data sets used in this study are not publicly available.
